# The Story of the Insane from Year to Year

**Published:** 1907-01-26

**Authors:** 


### THE STORY OF THE INSANE FROM YEAR TO YEAR.

EIGHTEENTH ANNUAL SERIES.

### THE ABERDEEN ROYAL ASYLUM.

ON the first of January the register contained the names of 882 patients, and on the thirty-first of December the number had fallen to 829. There were 147 first admissions; 40 readmissions; 77 discharged recovered; 22 discharged relieved; 72 discharged not improved; and 69 died. The total number under treatment was 1,069, and the average daily number resident was 844. The recoveries stand at 41.1 per cent. of the admissions; and the deaths at 8.1 per cent. of the average number resident. Of the year's deaths 28 were caused by cerebral and spinal diseases; 10 by lung diseases other than consumption, of which only 4 died. Of the year's admissions, 12 owed their insanity to domestic troubles, adverse circumstances, mental anxiety, religion and love. Hereditary influence accounted for 37; previous attacks for 27, and intemperance in drink for 23. The decrease in the numbers at the end of the year was caused by the transfer of 50 patients to the Aberdeen District Asylum; 23 were boarded out; and others were sent to other asylums in Scotland. The consequence of these removals is that the Royal Asylum will become more of an institution for private patients; in fact, nearly all the paupers chargeable to the City of Aberdeen were removed shortly after the date of the report; and the Committee seems a little anxious as to the effect which this sudden removal would have on their charges for maintenance of the remainder. One point on which Dr. Reid may be congratulated is that with their removal there have been no general paralytics sent to the asylum from other districts in the county. The charge for pauper patients is £32 per annum; for private patients from £30 to £365 per annum.

### CUMBERLAND AND WESTMORLAND ASYLUM.

IN this asylum the numbers increased during the year from 730 to 743. There were 124 first admissions, 32 readmissions, 68 were discharged recovered, 45 were discharged not improved, and 67 died. The total number under treatment during the year was 886, and the average resident was 739. Calculated on the admissions the recoveries stand at 43.5 per cent., and calculated on the average number resident, the deaths were 9 per cent. Of the year's deaths 15 were caused by cerebral and spinal diseases, 10 by consumption, and 4 by pneumonia. Moral causes such as domestic trouble and adverse circumstances account for the insanity in 26 of the admissions, and among the physical causes hereditary influence accounts for 51, previous attacks for 41, and drink for 17. Dr. Farquharson shows that heredity and drink are responsible for upwards of 42 per cent. of the admissions, and it is to be feared that even this high percentage is too low, because the friends of the insane very often do their best to conceal the hereditary taint and the drink habit. The inhabitants of Cumberland will be glad to learn that the county has a low proportion of the insane to its population—indeed it is only 2.5 per thousand, whereas the ratio for the whole of England is upwards of 3 per thousand. Dr. Farquharson emphasises the importance of early treatment in mental diseases by pointing out that excepting two patients, all the recoveries of the year took place in cases admitted to the asylum within twelve months of the onset of the attack. The asylum engineer retired during the year and was granted a pension of £70. The Committee say they are "well satisfied with the superintendence of the asylum." The weekly cost per head is 9s.

### LEICESTER BOROUGH ASYLUM.

ON the first of January this asylum contained 795 patients, of whom 326 were men and 469 women. There were 155 first admissions; 24 readmissions; 56 discharged recovered; 25 discharged not improved, and 70 deaths. The total number under treatment during the year was 968, and the average number resident was 797. The recoveries were 39.7 per cent. of the admissions, and the deaths were 8.7 per cent. of the average number resident. Of the year's deaths 28 were caused by cerebral and spinal diseases; 11 by consumption; 4 by pneumonia, and 4 by other lung diseases. Turning to Table X we find that 26 of the patients admitted owed their insanity to various moral causes, and as usual among the physical causes hereditary influence and drink head the list, the former with 40 and the latter with 28. Dr. Finch tabulates the numbers of patients chargeable to the borough for a great many years, and it seems that ten years since the Borough of Leicester had a total population of 198,500, and of this total 527 were insane and confined in the asylum. Last year the estimated population of the borough was 228,000, and the patients had risen to 637. Of course these figures are exclusive of those who are in workhouses or in private asylums, and it is probable that in future the annual increment of asylum lunatics will not be fewer than 15, and it is likely to be more. Owing to ill-health, the

Clerk of the Asylum resigned his post and was awarded a pension of £150. Attendants Augrave and Biggs also received superannuation allowances of £20 10s. and £27 6s. 8d. The Committee record "their appreciation of the excellent manner in which Dr. Finch and the officers of the institution have discharged their duties." The weekly rate of maintenance is 11s. 1d. per head.

